# Intratumoral spatial heterogeneity at non-contrast CT predicts histological grading of invasive pulmonary adenocarcinoma: a multicenter retrospective study

**DOI:** 10.1371/journal.pone.0341163

**Published:** 2026-02-02

**Authors:** Shize Qin, Sijia Zhou, Yongying Liu, Dan Su, Qing Jia, Yang Li, Guohong Shen, Xiufu Zhang

**Affiliations:** 1 Department of Radiology, Jiangjin Central Hospital of Chongqing, Jiangzhou Avenue, Dingshan Sub-district, Jiangjin District, Chongqing, China; 2 Department of Radiology, Chongqing General Hospital, Xingguang Avenue, Liangjiang New Area, Chongqing, China; 3 Department of Research and Development, Shanghai United Imaging Intelligence Co., Ltd., Yunjin Road, Xuhui District, Shanghai, China; 4 Department of Radiology, People’s Hospital of Chongqing Hechuan, Hechuan District, Chongqing, China; Hokkaido University: Hokkaido Daigaku, JAPAN

## Abstract

**Objectives:**

The International Association for the Study of Lung Cancer (IASLC) grading system is key to the prognosis and treatment of Invasive Pulmonary Adenocarcinoma (IPA). However, current radiomics and other radiological approaches poorly capture tumor heterogeneity, limiting predictive power. This study aimed to develop an interpretable CT-based model that predicts the histological grading of IPA by decoding its intratumoral spatial heterogeneity.

**Materials and methods:**

This multi‑center retrospective study enrolled 355 IPA patients, split into training/validation (7:3) and an independent test cohort. Tumors were graded as low‑grade (Ⅰ/Ⅱ) or high‑grade (Ⅲ) per IASLC criteria. Intratumoral subregions were generated via unsupervised clustering of CT images, and their spatial interaction heterogeneity was quantified using a Multi-regional Spatial Interaction (MSI) matrix. Five models (clinical‑radiological, radiomics, MSI, radiomics‑combined, MSI‑combined) were built using four preprocessors and five classifiers. The optimal model was selected based on the Receiver Operating Characteristic (ROC) curve in the validation cohort, with generalizability assessed in the test cohort. Performance was compared via the DeLong test, and SHapley Additive exPlanations (SHAP) analysis interpreted feature contributions.

**Results:**

Three subregions were generated. The high-grade group exhibited a larger proportion of Subregion 1, while showing a smaller proportion of Subregion 2. The MSI model based on 10 MSI features achieved an AUC of 0.806 in the test cohort, outperforming clinical‑radiological, radiomics, and radiomics‑combined models (p = 0.002, 0.010, 0.022). Adding clinical‑radiological features did not improve the MSI model (p = 0.083). SHAP identified MSI_border_proportion_2_3 (relative border proportion between Subregions 2 and 3) as the most influential feature, with lower values indicating high‑grade IPA.

**Conclusion:**

The CT-based MSI model can predict the histological grade of IPA by decoding the spatial interaction heterogeneity of different subregions in the tumor, thereby providing reliable imaging evidence for preoperative individualized risk assessment.

## Introduction

Adenocarcinoma is the most common lung cancer subtype [1]. Invasive Pulmonary Adenocarcinoma (IPA) is more malignant and has a poorer prognosis than either adenocarcinoma in situ or minimally invasive adenocarcinoma [2,3]. Establishing a histological grading system is critical for evaluating the malignancy grade of IPA. Therefore, in 2020, the International Association for the Study of Lung Cancer (IASLC) proposed a grading system for IPA, which is based on the most predominant histological subtype and the proportion of high-grade components [4]. The IASLC grading system not only serves as an independent predictor of Overall Survival (OS) and Recurrence-Free Survival (RFS) in IPA but also informs clinical decision-making. It aids in identifying candidates for preoperative neoadjuvant therapy [5–7]. Furthermore, for patients who undergo surgery, the grading system provides a critical rationale for postoperative adjuvant chemotherapy, as evidenced by studies demonstrating superior outcomes in high-grade (Grade 3) stage Ib-III patients receiving such treatment [8,9]. Therefore, the accurate preoperative assessment of IASLC grade is crucial for predicting prognosis and formulating optimal treatment strategies in patients, specifically determining the extent of resection (lobectomy or segmentectomy) and deciding whether to use neoadjuvant/adjuvant therapy.

The minimally invasive biopsies with high diagnostic yield are generally considered the preferred option for the preoperative diagnosis of lung adenocarcinoma. However, preoperative biopsy is limited by tumor heterogeneity and insufficient sample size, making a comprehensive assessment of histological grades challenging [10]. CT imaging holds significant value in diagnosing the malignancy grade and pathological subtypes of lung cancer [11,12]. A higher IASLC grade of IPA was associated with more invasive features on CT, suggesting that CT imaging can help characterize tumor grade [13,14]. However, due to the diversity and overlap of CT features in IPA—coupled with the fact that assessment results are prone to being influenced by physicians’ subjective experience—these factors can all lead to suboptimal predictive performance.

Radiomics enables the extraction of a series of high-dimensional quantitative features from medical images, thereby helping reveal underlying imaging patterns imperceptible to the naked eye [15]. In recent years, it has been applied to histological grading, invasiveness prediction, etc., of IPA [16,17]. However, conventional radiomics analysis methods often treat tumors as homogeneous wholes and overlook intratumoral subregional variations; this practice ultimately leads to the masking of local heterogeneity information. To address this limitation, a new analytical method is required: it employs algorithms (e.g., unsupervised clustering) to partition voxels with similar imaging features into distinct subregions, which may exhibit unique growth and invasion patterns. This approach not only overcomes the drawbacks of conventional radiomics but also better captures intratumoral heterogeneity [18–20]. For instance, an intratumoral heterogeneity score derived from subregion analysis has proven valuable for distinguishing histological subtypes in lung adenocarcinoma presenting as pure ground-glass nodules [21] and for predicting high-grade patterns in solid lung adenocarcinoma [22]. Furthermore, extending this concept to a three-dimensional space incorporating multiple perspective heterogeneity metrics has demonstrated superior performance in preoperative IASLC grading [23]. The aforementioned studies have effectively characterized tumor heterogeneity by quantifying subregional attributes and their spatial distribution. To further capture the potential spatial interactions among subregions, the Multi-regional Spatial Interaction (MSI) matrix is introduced to quantify the adjacency and spatial co-occurrence relationships of subregions in three-dimensional space, thereby providing a complementary characterization of tumor spatial heterogeneity [24]. Previous studies have established that MSI matrix-derived spatial heterogeneity features are independent prognostic factors for OS and RFS in Non-Small Cell Lung Cancer (NSCLC) [25]. However, the relationship between this specific form of spatial heterogeneity, quantified by the MSI matrix, and the histological grading of IPA remains to be fully investigated.

Therefore, this study aims to investigate the predictive value of spatial heterogeneity quantified by the combined approach of density-based subregion analysis and MSI matrix for the histological grades of IPA. Our goal is to provide valuable supplementary information to pathological diagnosis from an imaging perspective, ultimately to assist in improving preoperative grading accuracy.

## Materials and methods

### Patients

This retrospective study was conducted in accordance with the Declaration of Helsinki and received approval from the Institutional Review Boards of all participating hospitals: the Ethics Committee of Jiangjin Central Hospital of Chongqing (Center 1, KY20250619−002), the Ethics Committee of Chongqing General Hospital (Center 2, IIT-S2025-041–01), and the Ethics Committee of the People’s Hospital of Chongqing Hechuan (Center 3, HX-2025-020-001). We retrospectively collected data from 599 patients with pathologically confirmed lung adenocarcinoma who underwent surgical resection between June 2022 and February 2025. The data were accessed for research purposes on August 1, 2025. During the initial data collection, the authors had access to identifying information (including patient names and medical record numbers); however, all such identifiers were permanently removed and replaced with anonymous study codes before statistical analysis to ensure confidentiality. The requirement for informed consent was waived. To avoid clustering effects, only one largest lesion was selected for subsequent analysis in patients with multiple lesions.

A sample size estimation was performed to ensure adequate statistical power for model development. The calculation was based on the standard formula for estimating a diagnostic proportion with a specified precision [26]:


$$N=\frac{Z_{\text{1}-\alpha /\text{2}}^{\text{2}}\times P(\text{1}-P)}{\Delta^{\text{2}}}$$

where ${\text{Z}}_{\text{1}-\alpha /\text{2}}$=1.96 corresponds to a two-sided significance level of α=0.05, P is the expected proportion (sensitivity or specificity), and Δ is the allowable error (set at 0.08). Using expected sensitivity (80%) and specificity (70%) as reference, the minimum required sample size was calculated to be 224.

Inclusion Criteria: (1) Chest CT scan performed within one month prior to surgery; (2) Availability of detailed postoperative pathological reports. Exclusion Criteria: (1) Poor-quality CT images; (2) History of preoperative treatment; (3) Pathological subtypes including minimally invasive adenocarcinoma, invasive mucinous adenocarcinoma, and other variants of adenocarcinoma (e.g., colloid adenocarcinoma, fetal adenocarcinoma, or enteric adenocarcinoma).

Ultimately, 355 patients with IPA were enrolled in this study. From Center 1 and Center 2, a total of 227 patients were randomly assigned to a 161 (n = 161) or a validation cohort (n = 66) in a 7:3 ratio. An additional 128 patients from Center 3 formed an independent test cohort.

**Fig 1:**
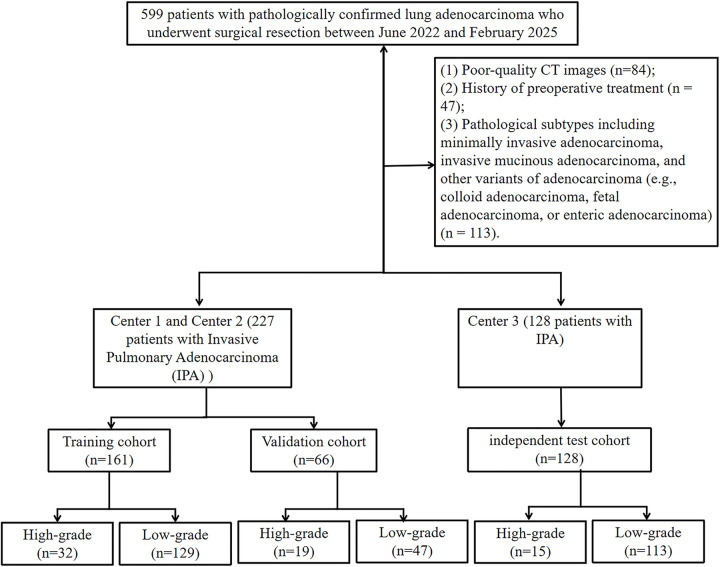
The flow chart of patient enrollment.

According to the IASLC grading system, grade 3 is defined as invasive adenocarcinoma with a total proportion of high-grade patterns (solid, micropapillary, or complex glandular patterns) of 20% or more, irrespective of the predominant subtype. Grade 3 IPA is associated with a poorer prognosis and benefits from adjuvant chemotherapy, demonstrating significant differences compared to grade 1 and 2 tumors [4,5,7]. Based on this, we stratified the enrolled patients into a low-grade group (grades 1 and 2) and a high-grade group (grade 3). The flowchart of patient enrollment is shown in Fig 1.

### CT imaging protocol

Non-enhanced chest CT scans were performed at each center using the following scanners: Center 1 utilized the United Imaging UCT530 40-slice CT scanner (United Imaging Healthcare, Shanghai, China), Center 2 employed the Philips IQon Spectral 64-slice CT scanner (Philips Medical Systems, Best, The Netherlands), and Center 3 used the Philips Brilliance iCT 128-slice scanner. Detailed scanning parameters for all three centers are provided in S1 Table.

### Clinical and radiological characteristics

Patient baseline characteristics, including gender, age, smoking history, alcohol consumption, and clinical stages, were recorded using the electronic medical record system. Two radiologists with 10 and 7 years of experience in thoracic imaging, respectively, independently and blindly evaluated the following radiological characteristics: involved lobe, diameter, shape, attenuation, lobulation, air bronchogram, spiculation, pleural tag, and cavity. Any discrepancies between the two readers were resolved by consensus.

### Tumor segmentation

Tumor regions were automatically segmented following image preprocessing using the deep learning model VB-Net within the uAI Research Portal (version: 20240730). The model achieved an average Dice Similarity Coefficient (DSC) of 91.5% in delineating pulmonary nodules [27]. The two-dimensional slices were reconstructed into three-dimensional volumes of interest (VOIs). Subsequently, two radiologists with 8 and 15 years of work experience respectively performed layer-by-layer independent correction on the VOIs. The inter-observer DSC was 0.873. For regions with high inter-observer consistency (DSC ≥ 0.700), the segmentation results were adopted from the radiologist with more extensive experience (15 years of practice).

### Radiomic feature extraction

Before radiomic feature extraction, CT images underwent standardized preprocessing, which included the following steps: signal intensity normalization through gray-level discretization (bin width: 25); spatial resampling of voxels to an isotropic resolution of 1 mm × 1 mm × 1 mm using B-spline interpolation; and window setting normalization with a window width of 1500 HU and a level of −600 HU. Fourteen imaging filters were applied to the images for post-processing. Features were subsequently extracted from both the original and filtered image sets. A total of 2264 radiomic features were obtained from each VOI, comprising 104 original features, 432 gray-level statistical features, and 1728 textural features.

### Density-based subregion analysis

The original CT images in NIfTI format were loaded using SimpleITK. The window width and level were normalized to 1500 HU and −600 HU, respectively. Subsequently, the voxel values were scaled to the range [0, 1] using max-min normalization. The subregion segmentation was conducted in two steps. First, simple linear iterative clustering was applied to the voxel values within each VOI to perform supervoxel segmentation at the individual level (n_segments = 100). Second, a Gaussian mixture model was employed to cluster supervoxels with similar imaging phenotypes across the cohort, thereby identifying distinct subregions. The optimal number of subregions was determined based on cluster validity indices. The same criteria and quantitative procedures were applied to both the validation and independent test cohorts to obtain consistent subregion segmentation results.

Subsequently, an MSI matrix was constructed to characterize the spatial interaction heterogeneity by recording the co-occurrence relationships among subregions. First-order and second-order statistical features were then derived from the MSI matrix. The methodology for constructing the MSI matrix and the interpretation of the corresponding features are described in the S2 Text and S3 Fig.

### Feature selection

To address the issue of class imbalance, the synthetic minority oversampling technique (SMOTE) was applied exclusively to the training cohort to generate synthetic samples for the minority class, with concurrent down-sampling of the majority class (parameters: perc. over = 200, perc. under = 155).

All feature processing and selection steps described below were performed strictly within the training cohort. To eliminate scale differences, both the MSI and radiomic features were standardized separately using Z-score normalization. For each feature set, recursive feature elimination was then applied to remove redundant and non-discriminative features, followed by the application of Least Absolute Shrinkage and Selection Operator (LASSO) regression to select the most predictive subset. During LASSO modeling, 10-fold cross-validation was used to determine the optimal regularization parameter α based on the minimal cross-validation error for each feature set. Univariate and multivariate logistic regression analyses of clinical-radiological characteristics were performed to identify predictors independently associated with grading.

### Model construction

All models were trained and their hyperparameters were tuned using a uniform pipeline, and these processes were performed exclusively in the training cohort. Given the unknown distributional characteristics of the dataset—such as non-normality, outliers, and scale variations—and considering that different classifiers rely on distinct data assumptions, we adopted a comprehensive modeling strategy. This approach combined four preprocessing methods (Box-Cox transformer, MaxAbs scaling, Quantile transformer, and Yeo-Johnson transformer) with five classifiers (Random Forest (RF), Logistic Regression (LR), Quadratic Discriminant Analysis (QDA), Support Vector Machine (SVM), and Decision Tree (DT)) to identify the optimal model for the current dataset. Based on the selected clinical-radiological, MSI, and radiomic features, we constructed three distinct models (clinical-radiological, MSI, and radiomics) and two integrated models: an MSI-combined model (incorporating clinical-radiological and MSI features) and a radiomics-combined model (incorporating clinical-radiological and radiomic features).

### Model performance evaluation and interpretability analysis

The validation cohort was used solely to select the optimal model, while the independent test cohort was employed strictly to evaluate the model’s generalization performance. Receiver Operating Characteristic (ROC) curves were plotted, and the Area Under the Curve (AUC) was calculated to evaluate the models’ ability to discriminate between different grades. Calibration curves were generated along with the Brier score to assess the agreement between predicted probabilities and actual outcomes. Decision Curve Analysis (DCA) was applied to quantify the net clinical benefit. DeLong’s test was used to compare AUC values among different models.

The model’s decision-making process was interpreted using SHapley Additive exPlanations (SHAP; version 0.46.0) to visualize feature contributions and enhance clinical usability.

### Statistical analysis

Normality of continuous variables was assessed using the Kolmogorov-Smirnov test. Data conforming to a normal distribution are presented as mean ± standard deviation (Mean ± SD), and group comparisons were performed using the independent samples t-test. Non-normally distributed data are expressed as median (interquartile range) [M (Q1, Q3)], and the Mann-Whitney U test was used for intergroup comparisons. Categorical variables are summarized as frequency (percentage) [n (%)], and group differences were assessed using the chi-square test. The statistical significance level was set at *p* < 0.05. All statistical analyses were conducted using the uAI Research Portal and SPSS statistical software (version 26.0; IBM, Armonk, NY, USA).

## Results

### Clinical-radiological characteristics and clinical-radiological model construction

**Fig 2:**
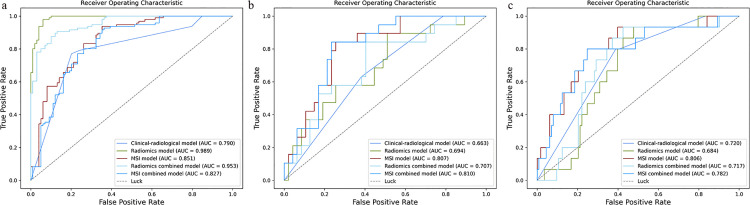
Performance of the five developed models. **a** Training cohort, **b** validation cohort, and **c** independent test cohort.

This study included a total of 355 patients with IPA, among whom 66 (18.6%) were classified into the high-grade group. The clinical-radiological characteristics of the three cohorts are summarized in Table 1. Most baseline characteristics showed no significant differences across the cohorts, with the exception of clinical stages (*p* < 0.001). Multivariable logistic regression analysis identified nodule/mass attenuation (*p* < 0.001) and lobulation (*p* = 0.025) as independent radiologic characteristics (Table 2). Among the clinical-radiological models constructed based on the above features, the combination of Box-Cox transformation and LR demonstrated the best predictive performance on the validation cohort and was therefore selected as the final model (S4 Table). This model achieved AUC values of 0.790, 0.633, and 0.720 in the training, validation, and independent test cohorts, respectively (Table 3; Figs 2a-2c).

**Table 1:** Clinical-radiological characteristics of patients in cohorts.

Characteristics		Training cohort				Validation cohort			Independent test cohort			*p*-inter
		Low-grade (n = 129)		High-grade (n = 32)	*p*-intra	Low-grade (n = 47)	High-grade (n = 19)	*p*-intra	Low-grade (n = 113)	High-grade (n = 15)	*p*-intra	
Gender (%)					0.002			0.218			0.549	0.223
	Male	38 (29.457)		19 (59.375)		17 (36.170)	10 (52.632)		51 (45.133)	8 (53.333)		
	Female	91 (70.543)		13 (40.625)		30 (63.830)	9 (47.368)		62 (54.867)	7 (46.667)		
Age (Median [Q25, Q75])		62.000 [56.000,70.000]		61.000 [53.750,68.250]	0.602	64.000 [58.000,69.000]	70.000 [55.500,72.000]	0.253	64.000 [56.000,69.000]	58.000 [55.500,66.500]	0.335	0.362
Smoking history (%)					0.011			0.035			0.906	0.148
	Long-term	19 (14.729)		12 (37.500)		8 (17.021)	7 (36.842)		23 (20.354)	2 (13.333)		
	Ever	36 (27.907)		4 (12.500)		19 (40.426)	2 (10.526)		12 (10.619)	1 (6.667)		
	Never	74 (57.364)		16 (50.000)		20 (42.553)	10 (52.632)		78 (69.027)	12 (80.000)		
Alcohol consumption (%)					0.454			0.037			0.571	0.264
	Heavy	16 (12.403)		3 (9.375)		8 (17.021)	5 (26.316)		15 (13.274)	3 (20.000)		
	Light	41 (31.783)		14 (43.750)		23 (48.936)	3 (15.789)		2 (1.770)	0 (0.000)		
	Never	72 (55.814)		15 (46.875)		16 (34.043)	11 (57.895)		96 (84.956)	12 (80.000)		
Clinical stages (%)					0.002			0.013			0.239	<0.001
	Ⅰ	85 (65.891)		11 (34.375)		42 (89.362)	11 (57.894)		94 (83.186)	11 (73.334)		
	Ⅱ	27 (20.930)		16 (50.000)		2 (4.255)	4 (21.053)		15 (13.274)	2 (13.333)		
	Ⅲ	17 (13.179)		5 (15.625)		3 (6.383)	4 (21.053)		4 (3.540)	2 (13.333)		
Involved lobe (%)					0.680			0.046			0.491	0.021
	Superior lobe of left lung	29 (22.481)	5 (15.625)			14 (29.787)	8 (42.105)		31 (27.434)	3 (20.000)		
	Inferior lobe of left lung	21 (16.279)	8 (25.000)			1 (2.128)	3 (15.789)		16 (14.159)	4 (26.667)		
	Superior lobe of right lung	42 (32.558)	10 (31.250)			11 (23.404)	5 (26.316)		39 (34.513)	7 (46.667)		
	Middle lobe of right lung	8 (6.202)	3 (9.375)			2 (4.255)	1 (5.263)		10 (8.850)	0 (0.000)		
	Inferior lobe of right lung	29 (22.481)	6 (18.750)			19 (40.426)	2 (10.526)		17 (15.044)	1 (6.667)		
Tumor diameter (Median [Q25, Q75])		17.300 [12.500, 22.000]	19.050 [14.525, 26.200]		0.063	19.000 [12.100, 27.300]	24.000 [19.200, 31.400]	0.021	14.500 [13.650, 23.800]	23.200 [19.600, 26.210]	0.010	0.225
Tumor shape (%)					0.298			0.385			0.045	0.223
	Round/oval	12 (9.302)	5 (15.625)			4 (8.511)	3 (15.789)		3 (2.655)	2 (13.333)		
	Irregular	117 (90.698)	27 (84.375)			43 (91.489)	16 (84.211)		110 (97.345)	13 (86.667)		
Nodule/mass attenuation (%)					0.000			0.043			0.021	0.05
	Solid	42 (32.558)	29 (90.625)			18 (38.298)	12 (63.158)		46 (40.708)	12 (80.000)		
	Part-solid	66 (51.163)	2 (6.250)			19 (40.426)	7 (36.842)		56 (49.558)	3 (20.000)		
	Ground-glass	21 (16.279)	1 (3.125)			10 (21.277)	0 (0.000)		11 (9.735)	0 (0.000)		
Lobulation (%)					0.012			0.640			0.133	0.135
	Yes	100 (77.519)	31 (96.875)			40 (85.106)	17 (89.474)		98 (86.726)	15 (100.000)		
	No	29 (22.481)	1 (3.125)			7 (14.894)	2 (10.526)		15 (13.274)	0 (0.000)		
Air bronchogram (%)					0.049			0.399			0.263	0.223
	Yes	37 (28.682)	15 (46.875)			17 (36.170)	9 (47.368)		55 (48.673)	5 (33.333)		
	No	92 (71.318)	17 (53.125)			30 (63.830)	10 (52.632)		58 (51.327)	10 (66.667)		
Spiculation (%)					0.000			0.002			0.153	0.135
	Yes	64 (49.612)	28 (87.500)			23 (48.936)	17 (89.474)		69 (61.062)	12 (80.000)		
	No	65 (50.388)	4 (12.500)			24 (51.064)	2 (10.526)		44 (38.938)	3 (20.000)		
Pleural tag (%)					0.007			0.239			0.145	0.135
	Yes	86 (66.667)	29 (90.625)			33 (70.213)	16 (84.211)		87 (76.991)	14 (93.333)		
	No	43 (33.333)	3 (9.375)			14 (29.787)	3 (15.789)		26 (23.009)	1 (6.667)		
Cavity (%)					0.214			0.361			0.037	0.223
	Yes	6 (4.651)	0 (0.000)			2 (4.255)	0 (0.000)		6 (5.310)	3 (20.000)		
	No	123 (95.349)	32 (100.000)			45 (95.745)	19 (100.000)		107 (94.690)	12 (80.000)		

**Table 2:** Univariable and multivariate logistic regression analysis for the association between clinical-radiological characteristics and IASLC grade.

Clinical-radiological	Univariate			Multivariate		
	Coefficient	OR (95% CI)	*p* value	Coefficient	OR (95% CI)	*P* value
Pleural tag	−0.960	0.383 (0.177-0.831)	0.015			
Gender	−1.118	0.327 (0.182-0.587)	0.001			
Smoking history	−0.472	0.624 (0.439-0.887)	0.009			
Lobulation	−2.413	0.090 (0.020-0.396)	0.001	−1.907	0.148 (0.028-0.788)	0.025
Spiculation	−1.345	0.260 (0.134-0.508)	<0.001			
Nodule/mass attenuation	−1.733	0.177 (0.100-0.311)	<0.001	−2.049	0.129 (0.066-0.253)	<0.001
Clinical stages	0.575	1.778 (1.181-2.676)	0.006			

OR, odds ratio; CI, confidence interval

**Table 3:** Performance of all prediction models.

Cohort/Model		Clinical-radiological	Radiomics model	MSI model	Radiomics-combined	MSI-combined
Training						
	AUC (95%CI)	0.793 (0.730-0.850)	0.989 (0.979-0.999)	0.851 (0.798-0.905)	0.953 (0.928-0.979)	0.827 (0.769-0.886)
	Acc	0.785 (0.722-0.837)	0.949 (0.908-0.972)	0.769 (0.705-0.823)	0.877 (0.823-0.916)	0.759 (0.694-0.814)
	Sen	0.771 (0.677-0.844)	0.958 (0.898-0.984)	0.802 (0.711-0.869)	0.875 (0.794-0.927)	0.781 (0.689-0.852)
	Spe	0.798 (0.708-0.865)	0.939 (0.874-0.972)	0.737 (0.643-0.814)	0.879 (0.800-0.929)	0.737 (0.643-0.814)
	PPV	0.787 (0.725-0.839)	0.939 (0.896-0.965)	0.748 (0.682-0.803)	0.875 (0.821-0.914)	0.743 (0.677-0.799)
	NPV	0.782 (0.692-0.852)	0.959 (0.899-0.984)	0.793 (0.700-0.864)	0.879 (0.800-0.929)	0.777 (0.682-0.849)
Validation						
	AUC (95%CI)	0.663 (0.593-0.788)	0.694 (0.554-0.834)	0.807 (0.700-0.914)	0.707 (0.568-0.845)	0.810 (0.705-0.914)
	Acc	0.636 (0.516-0.742)	0.652 (0.531-0.755)	0.773 (0.658-0.857)	0.727 (0.610-0.820)	0.773 (0.658-0.857)
	Sen	0.526 (0.317-0.727)	0.579 (0.363-0.769)	0.842 (0.684-0.945)	0.474 (0.273-0.683)	0.842 (0.684-0.945)
	Spe	0.681 (0.538-0.796)	0.681 (0.538-0.796)	0.745 (0.605-0.847)	0.830 (0.699-0.911)	0.745 (0.605-0.847)
	PPV	0.400 (0.290-0.521)	0.423 (0.311-0.543)	0.571 (0.451-0.684)	0.529 (0.411-0.645)	0.571 (0.451-0.684)
	NPV	0.780 (0.633-0.880)	0.800 (0.652-0.895)	0.921 (0.792-0.973)	0.796 (0.664-0.885)	0.921 (0.792-0.973)
Independent test						
	AUC (95%CI)	0.720 (0.619-0.821)	0.684 (0.573-0.795)	0.806 (0.688-0.924)	0.717 (0.600-0.835)	0.782 (0.651-0.913)
	Acc	0.633 (0.547-0.711)	0.688 (0.603-0.761)	0.758 (0.677-0.824)	0.695 (0.611-0.768)	0.711 (0.627-0.782)
	Sen	0.800 (0.548-0.930)	0.533 (0.301-0.752)	0.800 (0.648-0.930)	0.733 (0.480-0.891)	0.800 (0.648-0.930)
	Spe	0.611 (0.518-0.695)	0.708 (0.618-0.784)	0.752 (0.665-0.823)	0.690 (0.600-0.768)	0.699 (0.609-0.776)
	PPV	0.214 (0.152-0.293)	0.195 (0.136-0.272)	0.300 (0.257-0.384)	0.239 (0.174-0.320)	0.261 (0.208-0.343)
	NPV	0.958 (0.885-0.986)	0.920 (0.843-0.960)	0.966 (0.905-0.988)	0.951 (0.881-0.981)	0.963 (0.898-0.987)

AUC, Area under the receiver operating characteristic curve; Acc, Accuracy; Sen, Sensitivity; Spe, Specificity; CI, Confidence interval; PPV, Positive predictive value; NPV, Negative predictive value

### Radiomic feature and radiomics model construction

A total of 10 radiomic features were retained after selection (S5 Table). In the validation cohort, the combination of Yeo-Johnson transformation and LR yielded the best performance (S4 Table) and was therefore selected as the final model. This model achieved AUC values of 0.989, 0.694, and 0.684 in the training, validation, and independent test cohorts, respectively (Table 3).

### Subregion segmentation

**Fig 3:**
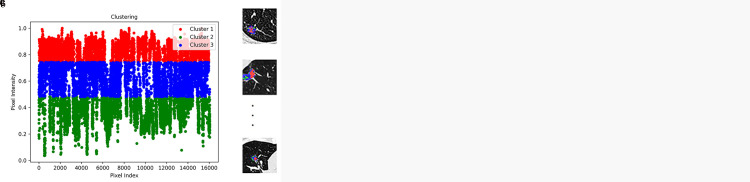
Tumor subregion segmentation was performed using a two-stage approach. **(a)** patient-level supervoxel generation using simple linear iterative clustering, followed by **(b)** cohort-level phenotypic clustering with a Gaussian mixture model. The optimal number of clusters (k = 3) was determined using the elbow method based on the rate of change in the sum of squared errors. The cohort-level clustering results are visualized in **(c)**. Color coding: red indicates Subregion 1, green represents Subregion 2, and blue corresponds to Subregion 3.

The optimal number of clusters was determined to be 3 based on the elbow method (Figs 3a-3c). The three density-based subregions exhibited distinct mean CT attenuation values. Subregion 1 exhibited a mean CT value of −72.6 HU, representing the component with the highest relative attenuation. This region is characterized by closely packed tumor cells, thickened alveolar septa, and minimal air content, corresponding to the denser, “sub-solid” portion of the tumor. Subregion 2 showed a mean CT value of −827.25 HU, representing the component with the lowest attenuation and predominantly consisting of air-filled alveolar structures and areas of sparse cellularity. Subregion 3 had a mean CT value of −424.05 HU, corresponding to the intermediate-attenuation component that consists of a mixture of “sub-solid” tissue and aerated structures with variable cellularity. The mean CT values of Subregion 1 (for which the *p*-value was 0.822), Subregion 2 (*p* = 0.255), and Subregion 3 (*p* = 0.838) did not differ significantly across the training, validation, and independent test cohorts, and this result supports the consistent mapping of CT density-based subregions across cohorts.

### MSI feature and MSI model construction

**Fig 4:**
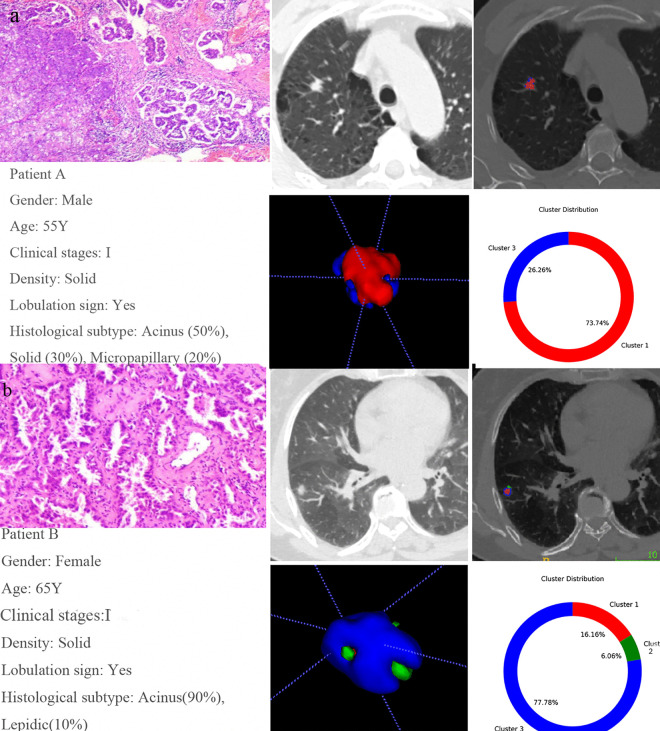
Comparison of tumor subregion proportions between high-grade and low-grade invasive pulmonary adenocarcinoma patients. Patient A (high-grade group): Subregion 1: 73.74%, Subregion 2: 0%, Subregion 3: 26.26% **(a)**. Patient B (low-grade group): Subregion 1: 16.16%, Subregion 2: 6.06%, Subregion 3: 77.78% **(b).**

**Fig 5:**
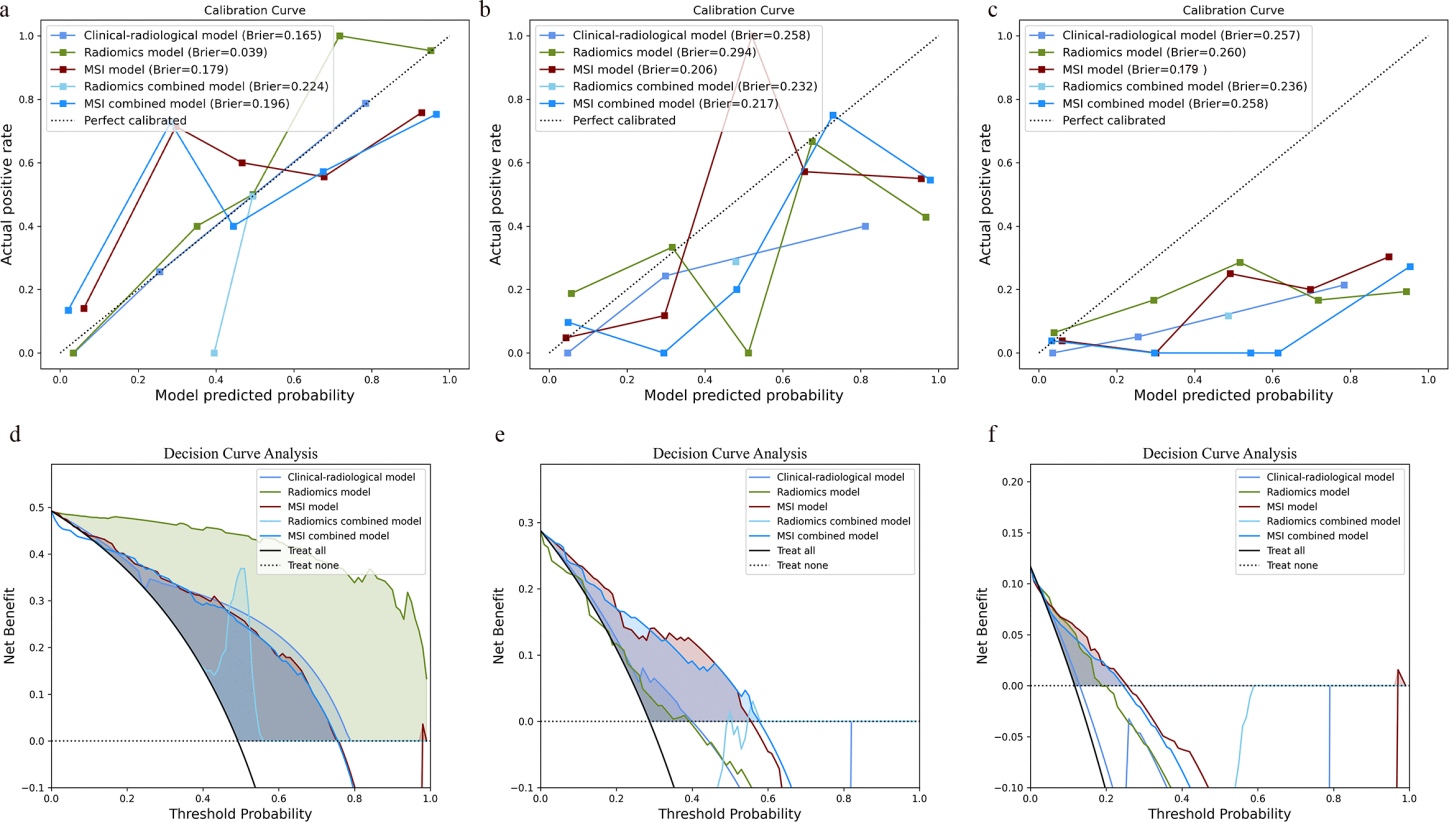
a-c and d-f depicts the calibration curve and decision curve analysis of the models in the training cohort, validation cohort, and independent test cohort, respectively.

A total of 33 first-order and 5 second-order statistical features were derived from the MSI matrix. The high-level group exhibited a substantially larger proportion of “Subregion 1” (median values of 0.667, 0.426, and 0.776 across the three cohorts), with broader interfaces to adjacent regions and the background matrix. In contrast, “Subregion 2” occupied a notably smaller proportion (median values of 0 across all cohorts) and demonstrated more limited boundary contact (S6 Table) (Figs 4a-4b). Ten MSI features were retained to construct the MSI model (S7 Table). The combination of Box-Cox transformation and QDA demonstrated the best performance in the validation cohort (S4 Table) and was therefore selected as the final model. This model achieved AUC values of 0.851, 0.807, and 0.806 in the training, validation, and independent test cohorts, respectively. In the independent test cohort, the MSI model exhibited superior predictive performance, significantly outperforming both the clinical-radiological model (*p* = 0.002) and the radiomics model (*p* = 0.010) (Table 4). Furthermore, the calibration curve indicated good agreement between predicted and actual probabilities, with a Brier score of 0.179 (Figs 5a-5c). DCA further confirmed that the MSI model provided higher net clinical benefit across a range of risk thresholds (Figs 5d-5f).

**Table 4:** Comparison of performance among models.

Cohort	Model	*p*-value
Validation		
	**MSI *vs.* Clinical-radiological**	**0.007**
	**MSI *vs.* Radiomics model**	**0.050**
	**MSI *vs.* Radiomics combined**	**0.042**
	**MSI** *vs.* MSI combined	0.882
	Radiomics *vs.* Clinical-radiological	0.615
	Radiomics *vs.* Radiomics combined	0.751
	Radiomics *vs.* MSI combined	0.065
	Clinical-radiological *vs.* Radiomics combined	0.440
	**Clinical-radiological *vs.* MSI combined**	**0.003**
	**Radiomics combined *vs.* MSI combined**	**0.047**
Independent test	**MSI *vs.* Clinical-radiological**	**0.002**
	**MSI *vs.* Radiomics model**	**0.010**
	**MSI *vs.* Radiomics combined**	**0.022**
	MSI *vs.* MSI combined	0.083
	Radiomics *vs.* Clinical-radiological	0.475
	Radiomics *vs.* Radiomics combined	0.356
	Radiomics *vs.* MSI combined	0.098
	Clinical-radiological *vs.* Radiomics combined	0.080
	Clinical-radiological *vs.* MSI combined	0.941
	Radiomics combined *vs.* MSI combined	0.128

We further integrated the independent radiological characteristics with the selected MSI and radiomic features to develop combined models. For the radiomics-combined model, the combination of MaxAbs Scaling and QDA demonstrated the best predictive performance (S4 Table), achieving AUC values of 0.953, 0.707, and 0.717 in the training, validation, and independent test cohorts, respectively. For the MSI-combined model, the optimal performance was attained using Box-Cox transformation combined with QDA (S4 Table), with corresponding AUC values of 0.827, 0.810, and 0.782 across the three cohorts. No statistically significant difference was observed between the predictive performance of the MSI-combined model and the MSI model (*p* > 0.05) (Table 4).

### Interpretability analysis of the MSI model

**Fig 6:**
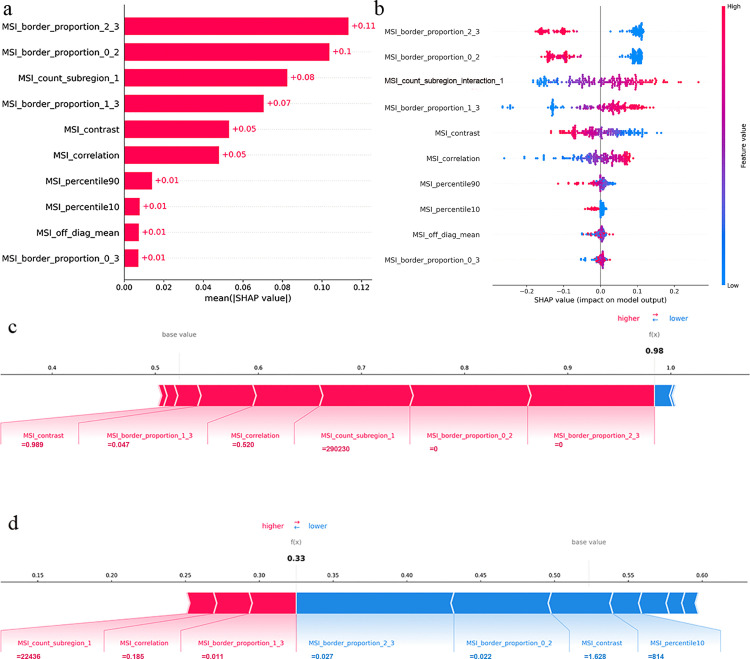
In the feature importance plot (a), the Y-axis shows features ranked by mean |SHAP| value (overall impact on prediction), with the most important feature at the top. The X-axis indicates mean |SHAP|; longer bars denote stronger feature influence. In the beeswarm plot (b), the X-axis displays individual SHAP values per sample. Red and blue dots represent high and low feature values, respectively. Force plots start from the base value. Each feature contributes a force proportional to its SHAP value, shown as an arrow: red increases the probability of high-grade prediction, blue decreases it. The sum of all forces yields the final prediction f(x). A 62-year-old female patient with pathology showing 30% micropapillary and complex glandular components (IASLC Grade 3). Model prediction (f(x)=0.980 > 0.525) classified it as high-grade, consistent with the pathological diagnosis (c). An 84-year-old male patient with pathology showing predominantly lepidic growth (IASLC Grade 1). Model prediction (f(x)=0.330 < 0.525) classified it as low-grade, consistent with the pathological diagnosis (d).

At the global level, the feature importance ranking (Fig 6a) revealed that MSI_border_proportion_2_3 was the most influential feature. This feature represents the relative border proportion between subregion 2 and 3. The beeswarm plot (Fig 6b) shows that higher values of MSI_border_proportion_2_3 correspond to negative SHAP values, indicating a lower likelihood of high-grade classification.

At the individual level, the SHAP force plots visually demonstrate the decision-making rationale of the MSI model for two representative patients (Figs 6c-6d).

### Subgroup analysis of the MSI model in different tumor sizes

The MSI model was employed to assess predictive performance across tumor size subgroups stratified as ≤20 mm, > 20 to ≤30 mm, and >30 mm. This classification is based on TNM staging thresholds and also ensures adequate subgroup sample sizes for comparative analysis. In the independent test cohort, the MSI model exhibited the highest diagnostic performance for IPA graded as >20 to ≤30 mm, with an AUC of 0.833 (95% CI: 0.690–0.976). This was followed by the diagnostic performance for IPA > 30 mm, which had an AUC of 0.822 (95% CI: 0.610–1.000). In contrast, the model showed relatively lower diagnostic performance for IPA ≤ 20 mm, with an AUC of 0.658 (95% CI: 0.363–0.954).

## Discussion

This study integrated CT density-based subregion analysis and the MSI matrix to quantify the spatial heterogeneity of IPA, and further explored this heterogeneity’s association with IASLC grading. Results showed that the MSI model exhibited the optimal performance; SHAP analysis revealed that MSI_border_proportion_2_3 (relative border proportion of subregion 2 to subregion 3) was the most important feature. From the perspective of spatial heterogeneity, this study initially achieved preoperative prediction of IASLC grading, which can provide pathologists with auxiliary diagnostic evidence.

IPA is a tumor with high heterogeneity, and its typical characteristic often involves a mixture of two or more histological subtypes [28]. However, traditional radiomics methods typically extract features by treating the entire tumor as a uniform region, thereby overlooking the differences between intratumoral subregions [11]. In fact, a tumor is a complex system comprising multiple subregions with distinct microenvironmental differences. Each subregion may correspond to different environmental selection pressures and cellular evolutionary paths. Independent analysis of these subregions allows for more precise characterization of the tumor’s heterogeneity, thereby revealing its biological behavioral characteristics more authentically [29]. Previous studies [30,31] have employed subregion analysis (e.g., via K-means clustering) to predict treatment response in cancers like nasopharyngeal carcinoma and NSCLC. However, these approaches have primarily focused on features extracted from individual subregions, with limited exploration of how these subregions are adjacency in three-dimensional space. In this study, we therefore introduced the MSI matrix, which aims to complement current methods by explicitly quantifying the adjacency and interaction patterns between subregions. Given that 3 subregions were identified in the study, a 4 × 4 MSI matrix (including 1 background region) was constructed. In the MSI matrix, diagonal elements reflect subregion volume, while off-diagonal elements quantify inter-subregion adjacency. We found a higher mean value of off-diagonal elements in high-grade tumors, indicating a greater overall quantity of interfaces between different subregions. This aligns with the pathological understanding that active proliferation and destructive infiltration in high-grade IPA disrupt the tissue architecture, resulting in a complex mixture of pathological components and blurred interregional borders [32]. The effectiveness of this method has been preliminarily verified in previous studies: Wu et al. [24] identified three subregions in breast cancer perfusion MR images and constructed an MSI matrix similarly. By using the derived features of the MSI matrix to perform risk stratification on patients, they found that this stratification was independently associated with RFS, confirming that imaging spatial heterogeneity has significant prognostic value.

Across the three subregions, Subregion 1 exhibited the highest mean CT attenuation, corresponding to the denser tissue component within the tumor. Previous studies [33,34] have linked elevated CT values to higher cellular density and desmoplastic stroma, which are histologic hallmarks of aggressive tumors. Therefore, Subregion 1 may correspond to more invasive pathological patterns, such as solid or micropapillary subtypes. Consistent with this, the higher proportion of Subregion 1 in our high-grade group aligns with the established pathological knowledge that high-grade IPA contains a greater proportion of these aggressive components. Notably, despite its association with denser tissue, the mean CT value of Subregion 1 was negative (−72.6 HU), which was lower than that of pure soft tissue (typically >0 HU). This phenomenon may be attributed to the influence of partial-volume averaging at tissue interfaces. Furthermore, in the MSI model, SHAP analysis identified two key predictive features: MSI_border_proportion_2_3 and MSI_border_proportion_0_2. Lower values of these features were associated with a higher likelihood of high-grade IPA. This observation is grounded in the pathological correlates of these subregions. Subregion 2, with very low CT attenuation, predominantly corresponds to air-containing structures or lepidic growth patterns, which represent indolent, non-invasive components. Subregion 3 exhibits intermediate attenuation, reflecting a mixture of denser tissue and aerated structures. Critically, the feature MSI_border_proportion_2_3 directly measures the extent of contact between these two components. A higher proportion implies greater connectivity between the lepidic/air-rich areas (Subregion 2) and the mixed-density tissue (Subregion 3), suggesting a tumor architecture that is more organized and less solid, a characteristic of a lower-grade, less invasive phenotype. Conversely, a lower proportion indicates that Subregion 3 has little direct contact with Subregion 2. In high-grade IPA, aggressive components expand as consolidated masses, pushing and displacing pre-existing lepidic structures, thereby minimizing this interface [35]. Consequently, our model captures a biomarker that reflects how tumor growth remodels internal architecture, yielding insights beyond simple measurements of component presence or volume.

This study also analyzed the overall spatial interaction heterogeneity of subregion distribution from the second-order statistics. Results showed that the high-grade group exhibited lower MSI_contrast and higher MSI_correlation. In our analysis, MSI_contrast quantifies the local differences in the strength of spatial interactions between adjacent subregions. A lower value indicates that the strength of spatial interactions between subregions is relatively homogeneous throughout the tumor. This likely mirrors the pathological feature of high-grade IPA, where high-grade components display a destructive, coalescent growth pattern [36]. This growth pattern blurs and intermingles the boundaries between different intra-tumoral “subregions”, rather than maintaining clearly separated compartments, and this characteristic is quantified as reduced contrast in the MSI matrix. MSI_correlation measures the linear dependency between row and column element values in the MSI matrix, which can be understood as the regularity or predictability of spatial interaction patterns across different local directions within the tumor. A higher value implies that, despite the intrinsic structural complexity of high-grade tumors, the spatial interaction patterns between subregions exhibit a certain degree of systematic and ordered arrangement in three-dimensional space. This observation is supported by prior research on tumor imaging phenotypes. In a study of NSCLC, Sujit et al. [25] similarly reported that a more aggressive disease state (high recurrence risk) was correlated with lower contrast and higher correlation. This further supports the close association between imaging spatial heterogeneity and the malignant behavior of tumors.

Among clinical-radiological characteristics, this study identified lobulation and attenuation of nodule/mass as independent predictors for grading, findings which are consistent with previous studies [16,37]. Lobulation sign and solid density are common invasive imaging features in high-grade IPA. However, when these CT features were further incorporated into the MSI model, the predictive performance of the model was not significantly improved. This result suggests that the features derived from the MSI matrix may have integrated key imaging information related to grading, which can reduce reliance on physicians’ subjective morphological interpretation to a certain extent.

This study has several limitations. First, the enrolled cohort had a relatively low rate of high-grade cases (18.6%), which may affect the stability of estimates for some metrics (e.g., yielding a wide confidence interval for AUC in subgroup analyses and a modest positive predictive value). Although we addressed class imbalance in the training cohort using SMOTE, the model’s performance in the independent test cohort mirrors this real-world challenge. Future studies with cohorts actively enriched for high-grade cases or large-scale multi-center collaborations are needed to further improve the precision of the model’s predictive performance. Second, subregion analysis was restricted to non-enhanced CT images—largely because non-enhanced CT was the most consistent and standardized protocol across all sites in this retrospective multi-center study. We note that contrast-enhanced CT could provide complementary information on tumor vascularity and heterogeneity; thus, while the current framework demonstrates the independent value of MSI features, it fails to capture all relevant tumor biological characteristics obtainable via multi-parametric imaging (e.g., contrast-enhanced CT, PET). Future work will integrate such multi-parametric data into subregion clustering to enhance the model. Third, translational application of the MSI model to routine clinical workflows faces practical challenges: (1) workflow integration: the automated segmentation/analysis pipeline (including subregion analysis and MSI matrix construction) needs embedding as a seamless plugin in existing PACS/RIS systems for efficient “one-click” DICOM processing; (2) clinical decision-making: the model’s probability output requires a clinically validated threshold (e.g., for guiding resection extent), which should be established via multidisciplinary consensus and net clinical benefit assessment; (3) clinician adoption: an intuitive reporting system that integrates quantitative predictions with visually clear, interpretable insights—for example, by highlighting key SHAP-based features—is essential to help clinicians correlate these outputs with conventional imaging assessment. In future studies, prospective multi-center validation and the development of a user-friendly, DICOM-compliant application will be undertaken to further confirm the model’s utility across diverse clinical settings.

## Conclusions

This study demonstrates that the CT-based MSI model can predict histological grading of IPA by decoding the spatial interaction heterogeneity of different subregions in the tumor. By leveraging SHAP analysis to elucidate feature contributions, we enhanced the model’s interpretability and clinical application. These findings offer a valuable supplement to pathological diagnosis, providing objective imaging evidence to guide preoperative risk stratification and personalized treatment planning.

## Supporting information

S1 Table CT scan parameters in center 1, center 2, and center 3. (DOCX)

S2 Text MSI matrix construction and feature interpretation. This file provides a detailed, step-by-step description of the methodology for constructing the MSI matrix, along with the specific mathematical interpretations of each derived MSI feature used in the MSI model.
(DOCX)

S3 Fig Schematic illustration of the MSI matrix construction. Shown in sequence: (a) The mapping of the three density-based subregions (and the background region) to a 4 × 4 matrix; (b) The meaning of diagonal/off-diagonal elements; (c) Types and quantities of the extracted MSI features.
(TIF)

S4 Table The performance of the combination of model preprocessors and classifiers. (DOCX)

S5 Table Radiomics features selected by LASSO and their coefficients. (DOCX)

S6 Table MSI features of patients in cohorts. (DOCX)

S7 Table MSI features selected by LASSO and their coefficients. (DOCX)

S8 Dataset Anonymized raw clinical-radiological data. This CSV file contains the de-identified dataset of 355 patients, with age reported in groups.
(XLSX)

S9 Dataset Anonymized raw MSI features. (XLSX)

S10 Dataset Anonymized raw radiomics features. (XLSX)

S11 Text Data dictionary for S8 Dataset. This text file explains all variable names, categorical codes, and the anonymization procedure.
(TXT)
