# Supplementary material for: Intratumoral spatial heterogeneity at non-contrast CT predicts histological grading of invasive pulmonary adenocarcinoma: a multicenter retrospective study
Source: PLoS One. 2026 Feb 2;21(2):e0341163. doi: 10.1371/journal.pone.0341163 (PMC12863497; doi:10.1371/journal.pone.0341163)
Supplement: S1 Table — (DOCX) [file pone.0341163.s001.docx]

S1 Table CT scan parameters in center 1, center 2, and center 3

| Center | Tube voltage | Tube current | Pitch factor | Rotation time | Collimation width | Acquisition matrix | Reconstruction slice thickness/interval | Window width | Window center |
| --- | --- | --- | --- | --- | --- | --- | --- | --- | --- |
| 1 | 120KV | automatic tube current modulation (reference: 80 mAs) | 1.075 | 0.6 s/rev | 22 mm | 512×512 | 1mm/1mm | 1200 HU | -600 HU |
| 2 | 120KV | automatic tube current modulation (reference: 129 mAs) | 1.000 | 0.5 s/rev | 22 mm | 512×512 | 1mm/1mm | 1200 HU | -600 HU |
| 3 | 120KV | automatic tube current modulation (reference: 90 mAs) | 1.156 | 0.4 s/rev | 80 mm | 512×512 | 1mm/1mm | 1200 HU | -500 HU |
