# Supplementary material for: Intratumoral spatial heterogeneity at non-contrast CT predicts histological grading of invasive pulmonary adenocarcinoma: a multicenter retrospective study
Source: PLoS One. 2026 Feb 2;21(2):e0341163. doi: 10.1371/journal.pone.0341163 (PMC12863497; doi:10.1371/journal.pone.0341163)
Supplement: S2 Text — This file provides a detailed, step-by-step description of the methodology for constructing the MSI matrix, along with the specific mathematical interpretations of each derived MSI feature used in the MSI model. (DOCX) [file pone.0341163.s002.docx]

Materials and Methods

MSI Matrix Construction

Multi-regional spatial interaction (MSI) matrix was constructed to characterize the spatial interaction heterogeneity of subregions. This matrix was used to record the spatial co-occurrence statistical information among different subregions. The detailed implementation procedures can be grouped into three steps: (1) co-occurrence pairs within the local neighborhood of each voxel were identified and added to the corresponding cells of the MSI matrix; (2) this process was repeated until all voxels were processed; (3) the spatial distribution and the interaction of subregions were represented in the final generated MSI matrix. In the MSI matrices, the diagonal elements represent the number of intra-region connection, indicating the internal connectivity of each subregion, whereas the off-diagonal elements quantify the number of inter-region adjacencies, reflecting the spatial intersections between different subregions, reflecting the spatial interactions between them. First-order and second-order features were respectively extracted to quantize the extent and spatial distribution of intratumoral heterogeneity within multi-region maps.

MSI Feature Interpretation

| MSI features | Interpretation |
| --- | --- |
| First-order features (absolute counts based on MSI matrix) | |
| MSI_count_subregion_interaction_n | The total number of voxels in sub-region n and its interacting subregions. |
| MSI_border_0_n | The absolute border size (number of adjacency voxels) between background (0) and subregion (n). |
| MSI_border_n_1__n_2_ | The absolute border size between subregion (n_1_) and sub-region (n_2_). |
| MSI_mean, MSI_max | The mean, and maximum of the MSI matrix values, respectively. |
| MSI_variance, MSI_skewness, MSI_entropy and MSI_kurtosis | The variance, skewness, entropy, and kurtosis of the MSI matrix values, respectively. |
| MSI_percentile10/90 | The 10th percentile and 90th percentile of the MSI matrix values, respectively. |
| MSI_total_interactions | The total sum of the elements in the MSI matrix, representing the overall number of voxel-level interactions. |
| MSI_diag_mean, MSI_diag_std, and MSI_diag_ratio | The mean, standard deviation, and ratio of the diagonal elements in the MSI matrix, respectively. Note: The ratio is defined as the sum of the diagonal elements divided by the sum of all elements in the matrix. |
| MSI_off_diag_mean, MSI_off_diag_std, andMSI_off_diag_ratio | The mean, standard deviation, and ratio of the off-diagonal elements in the MSI matrix, respectively. Note: The ratio is defined as the sum of the off-diagonal elements divided by the sum of all elements in the matrix. |
| First-order features (relative counts based on normalized MSI matrix) | |
| MSI_proportion_subregion_n | The proportion of each subregion relative to the total. |
| MSI_border_proportion_0_n | The relative border proportion between background (0) and sub-region (n). |
| MSI_border_proportion_n_1__n_2_ | The relative border proportion between sub-region (n_1_) and sub-region (n_2_). |
| Second-order features (derived from the MSI matrix) | |
| dissimilarity, contrast, homogeneity, correlation, and energy | Summary statistics of spatial heterogenity of intratumor subregion maps based on MSI matrices. |
