# Supplementary figures and images for: Intratumoral spatial heterogeneity at non-contrast CT predicts histological grading of invasive pulmonary adenocarcinoma: a multicenter retrospective study
Source: PLoS One. 2026 Feb 2;21(2):e0341163. doi: 10.1371/journal.pone.0341163 (PMC12863497; doi:10.1371/journal.pone.0341163)

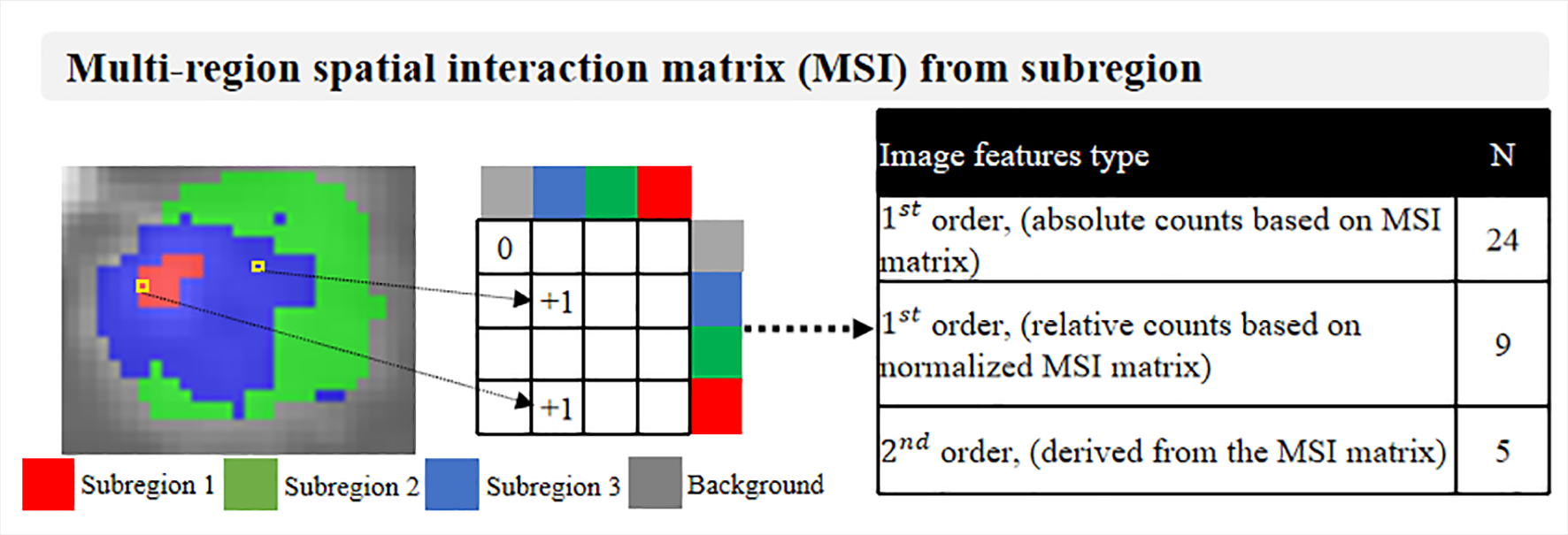

Supplement: S3 Fig — Shown in sequence: (a) The mapping of the three density-based subregions (and the background region) to a 4 × 4 matrix; (b) The meaning of diagonal/off-diagonal elements; (c) Types and quantities of the extracted MSI features. (TIF) [file pone.0341163.s003.tif]
