# Supplementary material for: Intratumoral spatial heterogeneity at non-contrast CT predicts histological grading of invasive pulmonary adenocarcinoma: a multicenter retrospective study
Source: PLoS One. 2026 Feb 2;21(2):e0341163. doi: 10.1371/journal.pone.0341163 (PMC12863497; doi:10.1371/journal.pone.0341163)
Supplement: S4 Table — (DOCX) [file pone.0341163.s004.docx]

S4 Table The performance of the combination of model preprocessors and classifiers

| Model | Training cohort | Validation cohort |
| --- | --- | --- |
| **Clinical-radiological model** |  |  |
|  | AUC (95%CI) | AUC (95%CI) |
| BoxCox_transformer>>DT | 0.819 (0.764-0.874) | 0.640 (0.509-0.771) |
| **BoxCox_transformer>>LR** | **0.793 (0.730-0.850)** | **0.663 (0.593-0.788)** |
| BoxCox_transformer>>RF | 0.818 (0.762-0.873) | 0.640 (0.509-0.771) |
| BoxCox_transformer>>SVM | 0.785 (0.723-0.846) | 0.663 (0.539-0.788) |
| BoxCox_transformer>>QDA | 0.786 (0.725-0.847) | 0.663 (0.539-0.788) |
| Max_abs_scaler>>DT | 0.819 (0.764-0.874) | 0.603 (0.461-0.745) |
| Max_abs_scaler>>LR | 0.786 (0.724-0.847) | 0.663 (0.593-0.788) |
| Max_abs_scaler>>RF | 0.818 (0.762-0.873) | 0.616 (0.475-0.758) |
| Max_abs_scaler>>SVM | 0.786 (0.724-0.847) | 0.663 (0.539-0.788) |
| Max_abs_scaler>>QDA | 0.786 (0.724-0.847) | 0.663 (0.539-0.788) |
| Quantitle_transformer>>DT | 0.819 (0.764-0.874) | 0.603 (0.461-0.745) |
| Quantitle_transformer>>LR | 0.790 (0.730-0.850) | 0.663 (0.539-0.788) |
| Quantitle_transformer>>RF | 0.818 (0.762-0.873) | 0.616 (0.475-0.758) |
| Quantitle_transformer>>SVM | 0.786 (0.724-0.847) | 0.663 (0.593-0.788) |
| Quantitle_transformer>>QDA | 0.790 (0.730-0.850) | 0.663 (0.539-0.788) |
| YeoJohnson_transformer>>DT | 0.819 (0.764-0.874) | 0.603 (0.461-0.745) |
| YeoJohnson_transformer>>LR | 0.785 (0.723-0.846) | 0.644 (0.515-0.774) |
| YeoJohnson_transformer>>RF | 0.818 (0.762-0.873) | 0.616 (0.475-0.758) |
| YeoJohnson_transformer>>SVM | 0.785 (0.723-0.846) | 0.663 (0.539-0.788) |
| YeoJohnson_transformer>>QDA | 0.785 (0.723-0.846) | 0.663 (0.539-0.788) |
| **Radiomics model** |  |  |
|  | AUC (95%CI) | AUC (95%CI) |
| BoxCox_transformer>>DT | 0.993 (0.987-1.000) | 0.684 (0.545-0.823) |
| BoxCox_transformer>>LR | 0.987 (0.974-1.000) | 0.692 (0.554-0.831) |
| BoxCox_transformer>>RF | 0.982 (0.969-0.995) | 0.655 (0.497-0.813) |
| BoxCox_transformer>>SVM | 0.986 (0.970-1.000) | 0.679 (0.536-0.821) |
| BoxCox_transformer>>QDA | 0.977 (0.962-0.992) | 0.684 (0.534-0.834) |
| Max_abs_scaler>>DT | 0.993 (0.987-1.000) | 0.676 (0.537-0.815) |
| Max_abs_scaler>>LR | 0.982 (0.969-0.996) | 0.660 (0.508-0.811) |
| Max_abs_scaler>>RF | 0.982 (0.969-0.995) | 0.677 (0.511-0.844) |
| Max_abs_scaler>>SVM | 0.967 (0.946-0.988) | 0.674 (0.525-0.823) |
| Max_abs_scaler>>QDA | 0.967 (0.946-0.988) | 0.671 (0.521-0.820) |
| Quantitle_transformer>>DT | 0.993 (0.987-1.000) | 0.662 (0.525-0.799) |
| Quantitle_transformer>>LR | 0.985 (0.975-0.996) | 0.679 (0.532-0.826) |
| Quantitle_transformer>>RF | 0.983 (0.970-0.995) | 0.650 (0.498-0.802) |
| Quantitle_transformer>>SVM | 0.981 (0.968-0.994) | 0.684 (0.538-0.831) |
| Quantitle_transformer>>QDA | 0.969 (0.950-0.988) | 0.664 (0.508-0.820) |

Supplementary Table 2 (continued)

| Model | Training cohort | Validation cohort |
| --- | --- | --- |
| YeoJohnson_transformer>>DT | 0.993 (0.987-1.000) | 0.685 (0.549-0.822) |
| **YeoJohnson_transformer>>LR** | **0.989 (0.979-0.999)** | **0.694 (0.554-0.834)** |
| YeoJohnson_transformer>>RF | 0.982 (0.969-0.995) | 0.645 (0.484-0.806) |
| YeoJohnson_transformer>>SVM | 0.990 (0.979-1.000) | 0.672 (0.526-0.818) |
| YeoJohnson_transformer>>QDA | 0.977 (0.691-0.992) | 0.669 (0.513-0.824) |
| **MSI model** |  |  |
|  | AUC (95%CI) | AUC (95%CI) |
| BoxCox_transformer>>DT | 0.961 (0.938-0.984) | 0.487 (0.333-0.640) |
| BoxCox_transformer>>LR | 0.918 (0.877-0.958) | 0.746 (0.622-0.870) |
| BoxCox_transformer>>RF | 0.924 (0.889-0.960) | 0.707 (0.581-0.834) |
| BoxCox_transformer>>SVM | 0.936 (0.900-0.973) | 0.728 (0.600-0.856) |
| **BoxCox_transformer>>QDA** | **0.851 (0.798-0.905)** | **0.807 (0.700-0.914)** |
| Max_abs_scaler>>DT | 0.961 (0.938-0.984) | 0.601 (0.470-0.731) |
| Max_abs_scaler>>LR | 0.899 (0.855-0.943) | 0.730 (0.599-0.862) |
| Max_abs_scaler>>RF | 0.924 (0.889-960) | 0.797 (0.691-0.904) |
| Max_abs_scaler>>SVM | 0.866 (0.813-0.919) | 0.754 (0.632-0.875) |
| Max_abs_scaler>>QDA | 0.820 (0.756-0.885) | 0.787 (0.672-0.903) |
| Quantitle_transformer>>DT | 0.961 (0.938-0.984) | 0.616 (0.471-0.762) |
| Quantitle_transformer>>LR | 0.899 (0.856-0.942) | 0.796 (0.684-0.908) |
| Quantitle_transformer>>RF | 0.925 (0.890-0.960) | 0.758 (0.641-0.874) |
| Quantitle_transformer>>SVM | 0.848 (0.795-0.901) | 0.791 (0.680-0.900) |
| Quantitle_transformer>>QDA | 0.796 (0.732-0.861) | 0.809 (0.702-0.915) |
| YeoJohnson_transformer>>DT | 0.961 (0.938-0.984) | 0.470 (0.327-0.612) |
| YeoJohnson_transformer>>LR | 0.912 (0.871-0.954) | 0.741 (0.612-0.871) |
| YeoJohnson_transformer>>RF | 0.924 (0.889-0.960) | 0.768 (0.646-0.889) |
| YeoJohnson_transformer>>SVM | 0.928 (0.890-0.966) | 0.730 (0.601-0.860) |
| YeoJohnson_transformer>>QDA | 0.848 (0.791-0.905) |  |
| **Radiomics-combined model** |  |  |
|  | AUC (95%CI) | AUC (95%CI) |
| BoxCox_transformer>>DT | 0.993 (0.987-1.000) | 0.684 (0.545-0.823) |
| BoxCox_transformer>>LR | 0.987 (0.974-1.000) | 0.697 (0.558-0.835) |
| BoxCox_transformer>>RF | 0.980 (0.966-0.994) | 0.641 (0.746-0.805) |
| BoxCox_transformer>>SVM | 0.986 (0.970-1.000) | 0.673 (0.529-0.817) |
| BoxCox_transformer>>QDA | 0.969 (0.950-0.988) | 0.680 (0.528-0.831) |
| Max_abs_scaler>>DT | 0.993 (0.987-1.000) | 0.676 (0.537-0.815) |
| Max_abs_scaler>>LR | 0.980 (0.965-0.994) | 0.682 (0.537-0.827) |
| Max_abs_scaler>>RF | 0.980 (0.966-0.994) | 0.660 (0.491-0.828) |
| Max_abs_scaler>>SVM | 0.961 (0.939-0.983) | 0.693 (0.552-0.835) |
| **Max_abs_scaler>>QDA** | **0.953 (0.928-0.979)** | **0.707 (0.568-0.845)** |
| Quantitle_transformer>>DT | 0.993 (0.987-1.000) | 0.662 (0.525-0.799) |
| Quantitle_transformer>>LR | 0.983 (0.971-0.995) | 0.683 (0.534-0.833) |
| Quantitle_transformer>>RF | 0.980 (0.966-0.994) | 0.642 (0.478-0.805) |

Supplementary Table 2 (continued)

| Model | Training cohort | Validation cohort |
| --- | --- | --- |
| Quantitle_transformer>>SVM | 0.974 (0.958-0.990) | 0.677 (0.522-0.832) |
| Quantitle_transformer>>QDA | 0.956 (0.932-0.980) | 0.683 (0.534-0.833) |
| YeoJohnson_transformer>>DT | 0.993 (0.987-1.000) | 0.685 (0.549-0.822) |
| YeoJohnson_transformer>>LR | 0.990 (0.980-1) | 0.693 (0.554-0.832) |
| YeoJohnson_transformer>>RF | 0.980 (0.966-0.994) | 0.633 (0.462-0.803) |
| YeoJohnson_transformer>>SVM | 0.989 (0.977-1.000) | 0.679 (0.533-0.824) |
| YeoJohnson_transformer>>QDA | 0.969 (0.950-0.987) | 0.682 (0.534-0.830) |
| **MSI-combined model** |  |  |
|  | AUC (95%CI) | AUC (95%CI) |
| BoxCox_transformer>>DT | 0.961 (0.938-0.984) | 0.487 (0.333-0.640) |
| BoxCox_transformer>>LR | 0.916 (0.874-0.958) | 0.720 (0.591-0.849) |
| BoxCox_transformer>>RF | 0.914 (0.875-0.953) | 0.721 (0.597-0.845) |
| BoxCox_transformer>>SVM | 0.933 (0.896-0.971) | 0.712 (0.580-0.845) |
| **BoxCox_transformer>>QDA** | **0.827 (0.769-0.886)** | **0.810 (0.705-0.914)** |
| Max_abs_scaler>>DT | 0.961 (0.938-0.914) | 0.601 (0.470-0.731) |
| Max_abs_scaler>>LR | 0.896 (0.850-0.942) | 0.727 (0.608-0.866) |
| Max_abs_scaler>>RF | 0.914 (0.874-0.953) | 0.768 (0.653-0.883) |
| Max_abs_scaler>>SVM | 0.873 (0.821-0.925) | 0.764 (0.647-0.880) |
| Max_abs_scaler>>QDA | 0.834 (0.774-0.893) | 0.792 (0.680-0.904) |
| Quantitle_transformer>>DT | 0.961 (0.938-0.984) | 0.616 (0.471-0.762) |
| Quantitle_transformer>>LR | 0.898 (0.855-0.942) | 0.765 (0.648-0.882) |
| Quantitle_transformer>>RF | 0.914 (0.876-0.953) | 0.750 (0.635-0.866) |
| Quantitle_transformer>>SVM | 0.868 (0.816-0.919) | 0.770 (0.657-0.884) |
| Quantitle_transformer>>QDA | 0.824 (0.765-0.884) | 0.798 (0.690-0.907) |
| YeoJohnson_transformer>>DT | 0.961 (0.938-0.984) | 0.470 (0.327-0.612) |
| YeoJohnson_transformer>>LR | 0.917 (0.877-0.957) | 0.713 (0.580-0.847) |
| YeoJohnson_transformer>>RF | 0.914 (0.875-0.953) | 0.777 (0.662-0.891) |
| YeoJohnson_transformer>>SVM | 0.928 (0.890-0.967) | 0.693 (0.554-0.833) |
| YeoJohnson_transformer>>QDA | 0.823 (0.763-0.884) | 0.792 (0.679-0.905) |

DT, Decision tree; LR, Logistic regression; RF, Random forest; SVM, Support vector machine; QDA, Quadratic discriminant analysis; AUC, Area under the receiver operating characteristic curve；Max_abs_scaler, MaxAbs Scaling
