# Supplementary material for: Intratumoral spatial heterogeneity at non-contrast CT predicts histological grading of invasive pulmonary adenocarcinoma: a multicenter retrospective study
Source: PLoS One. 2026 Feb 2;21(2):e0341163. doi: 10.1371/journal.pone.0341163 (PMC12863497; doi:10.1371/journal.pone.0341163)
Supplement: S5 Table — (DOCX) [file pone.0341163.s005.docx]

S5 Table Radiomics features selected by LASSO and their coefficients

| Type | Filter | Features | LASSO coefficient |
| --- | --- | --- | --- |
| Glszm | Specklenoise | SizeZoneNonUniformityNormalized | 0.192 |
| First-order | Normalize | Kurtosis | 0.133 |
| Glszm | Log_sigma_1.0_mm | SmallAreaHighGrayLevelEmphasis | 0.111 |
| Glszm | Curvatureflow | GrayLevelNonUniformity | 0.080 |
| Gldm | Wavelet_LHL | SmallDependenceLowGrayLevelEmphasis | 0.292 |
| First-order | Normalize | Mean | 0.006 |
| Glcm | Specklenoise | Correlation | -0.067 |
| Gldm | Mean | DependenceEntropy | -0.083 |
| Ngtdm | Wavelet_HLL | Strength | -0.095 |
| GLRLM | Additivegaussiannoise | ShortRunLowGrayLevelEmphasis | -0.135 |
