# Supplementary material for: Intratumoral spatial heterogeneity at non-contrast CT predicts histological grading of invasive pulmonary adenocarcinoma: a multicenter retrospective study
Source: PLoS One. 2026 Feb 2;21(2):e0341163. doi: 10.1371/journal.pone.0341163 (PMC12863497; doi:10.1371/journal.pone.0341163)
Supplement: S6 Table — (DOCX) [file pone.0341163.s006.docx]

S6 Table MSI features of patients in cohorts

| Features | Training cohort | |  | Validation cohort | |  | Independent test cohort | |  | *p*-inter |
| --- | --- | --- | --- | --- | --- | --- | --- | --- | --- | --- |
|  | low-grade  (n=129) | high-grade  (n=32) | *p*-intra | low-grade (n=47) | high-grade  (n=19) | *p*-intra | low-grade  (n=113) | high-grade  (n=15) | *p*-intra |  |
| MSI_count_subregion_interaction_1 (Median [Q1, Q3]) | 6894.000 [0.000,47006.000] | 93626.000 [25673.500,247241.000] | 0.000 | 5120.000 [0.000,23097.000] | 114046.000 [29393.000,289310.000] | 0.000 | 13526.000 [710.000,76356.000] | 220840.000 [89652.000,630381.000] | 0.000 | 0.489 |
| MSI_count_subregion_interaction_2 (Median [Q1, Q3]) | 10504.000 [0.000,45674.000] | 0.000 [0.000,0.000] | 0.000 | 8602.000 [729.000,56590.000] | 0.000 [0.000,15901.000] | 0.017 | 5326.000 [0.000,39642.000] | 0.000 [0.000,712.000] | 0.000 | 0.499 |
| MSI_count_subregion_interaction_3 (Median [Q1, Q3]) | 14366.000 [2864.000,52846.000] | 9945.000 [4353.000,26075.000] | 0.471 | 14930.000 [5567.000,43984.000] | 29170.000 [4884.000,137649.000] | 0.282 | 12252.000 [3684.000,43916.000] | 2934.000 [0.000,10022.000] | 0.015 | 0.119 |
| MSI_border_0_1 (Median [Q1, Q3]) | 614.000 [0.000,6502.000] | 11903.000 [4595.250,25258.500] | 0.000 | 662.000 [0.000,4303.500] | 13609.000 [3663.500,21087.500] | 0.000 | 2705.000 [65.000,11359.000] | 28124.000 [15528.000,53409.000] | 0.000 | 0.149 |
| MSI_border_0_2 (Median [Q1, Q3]) | 3501.000 [0.000,8841.000] | 0.000 [0.000,0.000] | 0.000 | 3202.000 [713.000,11158.000] | 0.000 [0.000,2614.000] | 0.009 | 1840.000 [0.000,9799.000] | 0.000 [0.000,0.000] | 0.000 | 0.542 |
| MSI_border_0_3 (Median [Q1, Q3]) | 3475.000 [427.000,9438.000] | 3464.500 [1308.000,6160.250] | 0.639 | 3451.000 [1062.000,8288.500] | 7415.000 [1858.000,18433.000] | 0.161 | 3168.000 [686.000,9484.000] | 36.000 [0.000,2308.500] | 0.008 | 0.262 |
| MSI_border_1_2 (Median [Q1, Q3]) | 0.000 [0.000,180.000] | 0.000 [0.000,0.000] | 0.041 | 0.000 [0.000,157.500] | 0.000 [0.000,243.500] | 0.871 | 32.000 [0.000,226.000] | 0.000 [0.000,4.000] | 0.047 | 0.107 |

Supplementary Table 4 (continued)

| Features | Training cohort | |  | Validation cohort | |  | Independent test cohort | |  | | *p*-inter | |
| --- | --- | --- | --- | --- | --- | --- | --- | --- | --- | --- | --- | --- |
|  | low-grade  (n=129) | high-grade  (n=32) | *p*-intra | low-grade  (n=47) | high-grade  (n=19) | *p*-intra | low-grade  (n=113) | high-grade  (n=15) | | *p*-intra | |  |
| MSI_border_1_3 (Median [Q1, Q3]) | 1173.000 [0.000,4752.000] | 3357.500 [1559.250,6821.000] | 0.001 | 1143.000 [0.000,3275.500] | 4623.000 [1499.500,14035.500] | 0.004 | 1436.000 [0.000,4369.000] | 912.000 [0.000,2824.500] | 0.367 | | 0.577 | |
| MSI_border_2_3 (Median [Q1, Q3]) | 1642.000 [0.000,3611.000] | 0.000 [0.000,0.000] | 0.000 | 1632.000 [235.500,5618.000] | 0.000 [0.000,4517.000] | 0.080 | 1542.000 [0.000,4915.000] | 0.000 [0.000,311.500] | 0.001 | | 0.234 | |
| MSI_proportion_subregion_1 (Median [Q1, Q3]) | 0.059 [0.000,0.355] | 0.667 [0.453,0.767] | 0.000 | 0.056 [0.000,0.241] | 0.426 [0.200,0.723] | 0.000 | 0.130 [0.010,0.542] | 0.776 [0.644,0.843] | 0.000 | | 0.358 | |
| MSI_proportion_subregion_2 (Median [Q1, Q3]) | 0.146 [0.000,0.505] | 0.000 [0.000,0.000] | 0.000 | 0.152 [0.012,0.558] | 0.000 [0.000,0.056] | 0.001 | 0.079 [0.000,0.401] | 0.000 [0.000,0.006] | 0.000 | | 0.791 | |
| MSI_proportion_subregion_3 (Median [Q1, Q3]) | 0.153 [0.052,0.326] | 0.082 [0.032,0.155] | 0.018 | 0.190 [0.072,0.340] | 0.244 [0.032,0.409] | 0.723 | 0.162 [0.041,0.306] | 0.008 [0.000,0.053] | 0.003 | | 0.122 | |
| MSI_border_proportion_0_1 (Median [Q1, Q3]) | 0.006 [0.000,0.044] | 0.066 [0.055,0.089] | 0.000 | 0.003 [0.000,0.024] | 0.043 [0.013,0.074] | 0.002 | 0.023 [0.001,0.077] | 0.076 [0.063,0.107] | 0.002 | | 0.054 | |
| MSI_border_proportion_0_2 (Median [Q1, Q3]) | 0.035 [0.000,0.106] | 0.000 [0.000,0.000] | 0.000 | 0.042 [0.008,0.097] | 0.000 [0.000,0.007] | 0.000 | 0.025 [0.000,0.085] | 0.000 [0.000,0.000] | 0.000 | | 0.896 | |

Supplementary Table 4 (continued)

| Features | Training cohort | |  | Validation cohort | |  | Independent test cohort | |  | *p*-inter |
| --- | --- | --- | --- | --- | --- | --- | --- | --- | --- | --- |
|  | low-grade  (n=129) | high-grade  (n=32) | *p*-intra | low-grade  (n=47) | high-grade  (n=19) | *p*-intra | low-grade  (n=113) | high-grade  (n=15) | *p*-intra |  |
| MSI_border_proportion_0_3 (Median [Q1, Q3]) | 0.028 [0.005,0.068] | 0.016 [0.005,0.052] | 0.352 | 0.035 [0.008,0.072] | 0.029 [0.008,0.060] | 0.713 | 0.034 [0.005,0.077] | 0.000 [0.000,0.010] | 0.005 | 0.689 |
| MSI_border_proportion_1_2 (Median [Q1, Q3]) | 0.000 [0.000,0.002] | 0.000 [0.000,0.000] | 0.046 | 0.000 [0.000,0.001] | 0.000 [0.000,0.002] | 0.835 | 0.000 [0.000,0.002] | 0.000 [0.000,0.000] | 0.045 | 0.153 |
| MSI_border_proportion_1_3 (Median [Q1, Q3]) | 0.007 [0.000,0.030] | 0.025 [0.007,0.052] | 0.003 | 0.005 [0.000,0.034] | 0.022 [0.009,0.046] | 0.066 | 0.011 [0.000,0.035] | 0.003 [0.000,0.008] | 0.076 | 0.888 |
| MSI_border_proportion_2_3 (Median [Q1, Q3]) | 0.019 [0.000,0.037] | 0.000 [0.000,0.000] | 0.000 | 0.025 [0.002,0.041] | 0.000 [0.000,0.013] | 0.003 | 0.014 [0.000,0.040] | 0.000 [0.000,0.003] | 0.002 | 0.640 |
| MSI_mean (Median [Q1, Q3]) | 6665.875 [3205.250,15453.250] | 9315.688 [5446.562,20942.094] | 0.049 | 6879.875 [3302.625,21019.062] | 19783.625 [8353.375,43611.500] | 0.019 | 9131.375 [3930.750,16798.750] | 17046.500 [8781.250,46762.188] | 0.014 | 0.309 |
| MSI_variance (Median [Q1, Q3]) | 199103396.609 [37497349.609,992517831.359] | 515851878.505 [144348867.516,3487686485.516] | 0.026 | 147995016.100 [31167564.922,1712208662.000] | 2069703674.734 [330870844.580,9982211460.297] | 0.017 | 252176253.400 [41992875.560,1286972888.000] | 2841749226.000 [461950381.300,23540054761.000] | 0.005 | 0.492 |

Supplementary Table 4 (continued)

| Features | Training cohort | |  | Validation cohort | |  | Independent test cohort | |  | *p*-inter |
| --- | --- | --- | --- | --- | --- | --- | --- | --- | --- | --- |
|  | low-grade (n=129) | high-grade  (n=32) | *p*-intra | low-grade (n=47) | high-grade  (n=19) | *p*-intra | low-grade (n=113) | high-grade  (n=15) | *p*-intra |  |
| MSI_max (Median [Q1, Q3]) | 58594.000 [22772.000,128786.000] | 93626.000 [48569.500,247241.000] | 0.020 | 49298.000 [22917.000,171274.000] | 189124.000 [71357.000,404903.000] | 0.017 | 65546.000 [24358.000,148020.000] | 220840.000 [89652.000,630381.000] |  |  |
| MSI_skewness (Median [Q1, Q3]) | 3.196 [2.410,3.627] | 3.731 [3.254,3.849] | 0.000 | 3.254 [2.433,3.590] | 3.398 [2.802,3.788] | 0.282 | 3.073 [2.438,3.605] | 3.763 [3.651,3.903] | 0.001 | 0.984 |
| MSI_kurtosis (Median [Q1, Q3]) | 11.214 [6.128,13.758] | 14.398 [11.419,15.099] | 0.000 | 11.370 [6.317,13.456] | 12.114 [8.536,14.723] | 0.395 | 10.461 [6.427,13.586] | 14.565 [13.896,15.423] | 0.001 | 0.993 |
| MSI_percentile10 (Median [Q1, Q3]) | 0.000 [0.000,120.000] | 0.000 [0.000,0.000] | 0.023 | 0.000 [0.000,91.500] | 0.000 [0.000,73.000] | 0.950 | 0.000 [0.000,168.000] | 0.000 [0.000,0.000] | 0.027 | 0.125 |
| MSI_percentile90 (Median [Q1, Q3]) | 13718.000 [6988.000,28702.500] | 16452.500 [10944.875,25609.500] | 0.337 | 13442.000 [6378.750,32175.500] | 35117.500 [16172.000,65063.750] | 0.015 | 16502.500 [8609.000,34387.000] | 28124.000 [16364.250,53409.000] | 0.035 | 0.235 |
| MSI_total_interactions (Median [Q1, Q3]) | 106654.000 [51284.000,247252.000] | 149051.000 [87145.000,335073.500] | 0.049 | 110078.000 [52842.000,336305.000] | 316538.000 [133654.000,697784.000] | 0.019 | 146102.000 [62892.000,268780.000] | 272744.000 [140500.000,748195.000] | 0.014 | 0.309 |
| MSI_entropy (Median [Q1, Q3]) | 2.237 [1.633,2.686] | 1.740 [1.192,2.327] | 0.015 | 2.342 [1.723,2.698] | 2.093 [1.451,2.510] | 0.240 | 2.417 [1.666,2.784] | 0.990 [0.828,1.824] | 0.001 | 0.812 |
| MSI_diag_mean (Median [Q1, Q3]) | 17452.500 [7530.000,44576.000] | 27426.500 [15258.375,67647.750] | 0.041 | 19656.000 [8176.750,59692.750] | 58377.000 [24896.250,141803.000] | 0.016 | 24505.000 [8834.500,48148.500] | 55210.000 [24880.500,158256.750] | 0.010 | 0.414 |

Supplementary Table 4 (continued)

| Features | Training cohort | |  | Validation cohort | |  | Independent test cohort | |  | *p*-inter |
| --- | --- | --- | --- | --- | --- | --- | --- | --- | --- | --- |
|  | low-grade  (n=129) | high-grade  (n=32) | *p*-intra | low-grade  (n=47) | high-grade  (n=19) | *p*-intra | low-grade  (n=113) | high-grade  (n=15) | *p*-intra |  |
| MSI_diag_std (Median [Q1, Q3]) | 23733.676 [9865.275,52185.945] | 38879.046 [20847.751,103299.441] | 0.021 | 20143.723 [9247.008,67422.248] | 77104.345 [29448.965,163777.172] | 0.015 | 25690.588 [10090.862,61166.979] | 95626.525 [37522.561,272586.079] | 0.003 | 0.526 |
| MSI_off_diag_mean (Median [Q1, Q3]) | 2141.500 [1342.625,4141.250] | 2711.812 [1813.062,4052.969] | 0.131 | 2512.500 [1259.688,4793.188] | 4695.750 [2129.312,7573.438] | 0.027 | 2655.500 [1569.250,5154.250] | 3629.500 [2823.625,6802.000] | 0.066 | 0.127 |
| MSI_off_diag_std (Median [Q1, Q3]) | 3601.610 [2169.543,5719.691] | 4367.367 [2915.018,8314.551] | 0.084 | 3647.366 [1905.753,8043.649] | 6742.772 [4332.105,12002.712] | 0.019 | 4541.348 [2388.784,7451.906] | 9262.862 [4974.609,17622.357] | 0.005 | 0.142 |
| MSI_diag_ratio (Median [Q1, Q3]) | 0.665 [0.600,0.740] | 0.731 [0.645,0.805] | 0.011 | 0.660 [0.575,0.742] | 0.761 [0.691,0.807] | 0.019 | 0.648 [0.578,0.755] | 0.776 [0.710,0.847] | 0.001 | 0.720 |
| MSI_off_diag_ratio (Median [Q1, Q3]) | 0.335 [0.260,0.400] | 0.269 [0.195,0.355] | 0.011 | 0.340 [0.258,0.425] | 0.239 [0.193,0.309] | 0.019 | 0.352 [0.245,0.422] | 0.224 [0.153,0.290] | 0.001 | 0.720 |
| MSI_dissimilarity (Median [Q1, Q3]) | 0.644 [0.463,0.850] | 0.398 [0.250,0.685] | 0.002 | 0.662 [0.448,0.888] | 0.443 [0.290,0.648] | 0.026 | 0.627 [0.401,0.836] | 0.224 [0.153,0.360] | 0.001 | 0.976 |
| MSI_contrast (Median [Q1, Q3]) | 1.399 [0.894,1.867] | 0.714 [0.325,1.550] | 0.003 | 1.472 [0.866,2.037] | 0.989 [0.452,1.545] | 0.041 | 1.369 [0.821,2.003] | 0.224 [0.164,0.539] | 0.001 | 0.821 |
| MSI_homogeneity (Median [Q1, Q3]) | 0.787 [0.741,0.839] | 0.848 [0.767,0.897] | 0.003 | 0.786 [0.720,0.850] | 0.845 [0.804,0.887] | 0.014 | 0.782 [0.734,0.845] | 0.888 [0.845,0.924] | 0.001 | 0.949 |
| MSI_correlation (Median [Q1, Q3]) | 0.067 [-0.056,0.194] | 0.195 [0.132,0.356] | 0.000 | 0.079 [-0.019,0.200] | 0.291 [0.072,0.450] | 0.011 | 0.084 [-0.053,0.193] | 0.098 [-0.083,0.226] | 0.865 | 0.390 |

Supplementary Table 4 (continued)

| Features | Training cohort | |  | Validation cohort | |  | Independent test cohort | |  | *p*-inter |
| --- | --- | --- | --- | --- | --- | --- | --- | --- | --- | --- |
|  | low-grade  (n=129) | high-grade  (n=32) | *p*-intra | low-grade  (n=47) | high-grade  (n=19) | *p*-intra | low-grade  (n=113) | high-grade  (n=15) | *p*-intra |  |
| MSI_energy (Median [Q1, Q3])) | 0.319 [0.223,0.469] | 0.468 [0.291,0.608] | 0.006 | 0.300 [0.205,0.450] | 0.347 [0.250,0.539] | 0.202 | 0.290 [0.203,0.455] | 0.628 [0.449,0.721] | 0.001 | 0.840 |
