# Supplementary material for: Intratumoral spatial heterogeneity at non-contrast CT predicts histological grading of invasive pulmonary adenocarcinoma: a multicenter retrospective study
Source: PLoS One. 2026 Feb 2;21(2):e0341163. doi: 10.1371/journal.pone.0341163 (PMC12863497; doi:10.1371/journal.pone.0341163)
Supplement: S7 Table — (DOCX) [file pone.0341163.s007.docx]

S7 Table MSI features selected by LASSO and their coefficients

| Features | LASSO coefficient | |
| --- | --- | --- |
| MSI_border_proportion_1_3 | 0.492 | |
| MSI_border_proportion_0_3 | 0.301 | |
| MSI_count_subregion_interaction_1 | 0.262 | |
| MSI_border_proportion_2_3 | 0.153 | |
| MSI_border_proportion_0_2 | 0.095 | |
| MSI_correlation | 0.078 |  |
| MSI_percentile90 | -0.030 |  |
| MSI_percentile10 | -0.086 |  |
| MSI_off_diag_mean | -0.300 |  |
| MSI_contrast | -0.758 |  |
